# Diet Selection by the Italian Hare (*Lepus corsicanus* de Winton, 1898) in Two Protected Coastal Areas of Latium

**DOI:** 10.3390/ani12060687

**Published:** 2022-03-09

**Authors:** Pierangelo Freschi, Simonetta Fascetti, Francesco Riga, Gabriella Rizzardini, Mario Fortebraccio, Marco Ragni, Rosanna Paolino, Carlo Cosentino

**Affiliations:** 1 School of Agricultural, Forestry, Food and Environmental Sciences (SAFE), University of Basilicata, 85100 Potenza, Italy; simonetta.fascetti@unibas.it (S.F.); gabriellarizzardini@virgilio.it (G.R.); fortebraccio.mario@gmail.com (M.F.); rosannapaol@gmail.com (R.P.); carlo.cosentino@unibas.it (C.C.); 2 Italian Institute for Environmental Protection and Research (ISPRA), 00144 Rome, Italy; francesco.riga@isprambiente.it; 3 Department of Agricultural and Environmental Science, University of Bari Aldo Moro, 70126 Bari, Italy; marco.ragni@uniba.it

**Keywords:** Italian hare, diet, micro-histological analysis, feeding preferences, ecological plasticity

## Abstract

**Simple Summary:**

In order to better understand the ecological niche of the Italian hare, we evaluated the diet selection of the species in two protected areas of the Latium coastal environment. The main results emerging from our study were: the wide feeding spectrum of the Italian hare; the high incidence of grasses in dry and in wet season diets; the low number of plant species ingested at relatively high rates; the plastic feeding behaviour of this hare, as diet preferences changed with the variety and abundance of food species. These results highlighted the great adaptability of the species to different niches and the influence of the floristic composition on its feeding habits. In the Italian hare, the assessment of habitat suitability is of strategic importance for its conservation. In particular, feeding preferences of the species may lead to defining some food items as key plant species for identifying its elective habitat and, hence, planning effective re-introduction initiatives.

**Abstract:**

This study was focused on the diet and feeding behaviour of *Lepus corsicanus* in two protected coastal areas of Latium, Castelporziano Presidential Estate (CPE) and Circeo National Park (CNP). Plant frequency was assessed by the quadrat method, while diet composition was determined by microhistological analysis of faecal samples. Over the year, the Italian hare fed on 185 of the 229 plant species identified in vegetation, with most of them ingested in low percentages (≤1%). During the dry season (DS), in both areas, *Brachypodium sylvaticum*, *Cynodon dactylon*, and *Avena fatua* were among the most consumed species. In the wet season (WS) the most common plant species in diet were *B. sylvaticum*, *Poa trivialis*, and *Carex distachya* in CPE and *Dactylis glomerata*, *Cynosurus echinatus*, and *Spartium junceum* in CNP. In both sites, considering the annual selection of life forms, grasses and leguminous forbs were preferred, while non-leguminous forbs and shrubs were used less than expected according to their availability. ANOSIM analysis showed significant differences between sites in DS and WS diets. Our study evidenced that the Italian hare behaved as generalist, revealing its capability for exploiting several plant species and to adapt its diet preferences to space-time variation of food availability.

## 1. Introduction

The areal extent of the Italian hare (*Lepus corsicanus* de Winton, 1898) covers central and southern Italy, Sicily, and Corsica but different size, density, and range of the Italian hare populations characterise each of these subareas [1]. In the peninsular area, the distribution of this endemic species has been subjected in the last decades to a substantial contraction accompanied by a significant reduction in the consistence of populations. The *taxon* recognizes as the northern limits the provinces of Grosseto, on the Tyrrhenian coast, and of Chieti, on the Adriatic side. In southern areas, the species is still present in all regions up to the Aspromonte National Park, but with relict populations, often isolated in protected or inaccessible mountainous areas [1,2]. In the peninsular subareal the most critical risk factors for the species are identified in the fragmentation of the distribution area, isolation and low population density, deterioration of the habitat, the introduction of the European hare (*Lepus europaeus* Pallas, 1788), and over-hunting [2]. On the contrary, in Sicily, the Italian hare is relatively widespread and is also observed in hunting areas far from protected parks. In Corsica, the presence of the species is evidenced in Haute-Corse and on the coastal area of Sagone, where the species is threatened by hybridization with *L. europaeus*, and with the Iberian hare (*Lepus granatensis* Rosenhauer, 1856) [3,4]. The species occupies mainly Mediterranean environments, even if it has been observed up to altitudes close to 2000 m [5]. In threatened species, such as the Italian hare, the assessment of habitat suitability is of strategic importance for their conservation. In particular, the listing of plants and their incidence in the Italian hares’ diet may lead to defining some food items as key plant species for identifying the elective habitat of the *taxon*, and hence planning an effective re-introduction initiative [5,6]. Additionally, the plants composing the diet may act as early warning indicators of food resource limitation, especially concerning diet overlap with other animals [7]. Studies on diet composition of the species, carried out in Sicily [8], Corsica [4], and in peninsular Italy [9,10,11,12,13], demonstrated that the Italian hare feeds on a large number of species of plants during the year, with a conspicuous presence of herbaceous ones (e.g., *B. sylvaticum*, *Trifolium pratense*, *Lolium arundinaceum*). Grasses and non-leguminous forbs represent the basis of the diet, with a higher incidence of Poaceae, Fabaceae, and Asteraceae in summer and of Rosaceae, Fagaceae, and Pinaceae (leaves, buds and barks) in the winter period [4,9,10,11].

Nevertheless, only little is known about the feeding preferences of the Italian hare and, among the aforementioned areas, only in Corsica, the feeding behaviour of this species was recently studied [4]. Knowledge of dietary selectivity in herbivores is a key element for the definition of their elective habitat and of the competition with other species [8,9,10,11,12,13]. In this study, in order to deepen this fundamental aspect of the trophic niche of *L. corsicanus*, we evaluated the effect of season on diet composition and feeding selection of the species in two protected areas of the Latium coastal environment in which there is no co-presence with *L. europaeus* [5]: Castelporziano Presidential Estate and Circeo National Park. In particular, the aims of this study were: (1) to analyse diet composition of the Italian hare in a Mediterranean habitat; (2) to provide a description of the use and selection of plant resources in accordance with their seasonal availability; (3) to identify key plant species in the diet; (4) to evaluate differences in diet composition between the periods using alpha and beta diversity indices.

## 2. Materials and Methods

### 2.1. Study Areas

**Figure 1:**
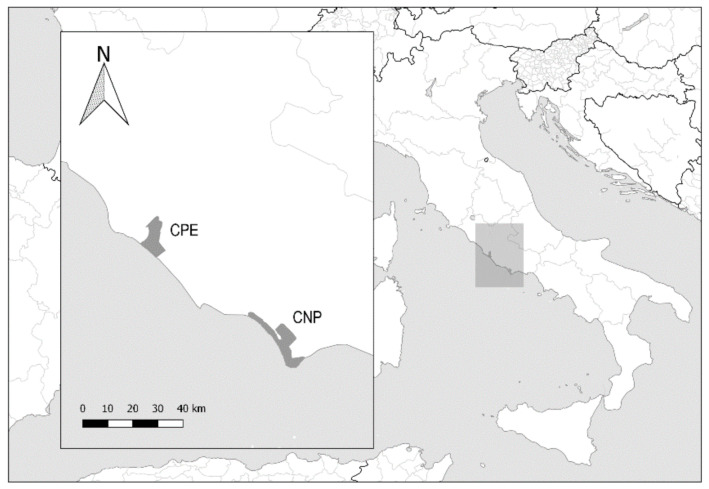
Map showing the study areas in Castelporziano Presidential Estate (CPE) and Circeo National Park (CNP) on the Latium coast.

Castelporziano Presidential Estate (CPE) is an enclosed and protected area that covers an area of about 5.892 ha (41°44′37.83″ N. 12°24′2.20″ E) (Figure 1). In this area, the annual means of temperature and precipitation are, respectively, +15.4 °C and 740 mm [6]. Circeo National Park (CNP) covering 8.917 ha (41°14′06″ N. 13°03′50.4″ E) is situated further South. Its mean annual rainfall is 963 mm with precipitation mainly concentrated in autumn and early winter (October–December) and the range of mean monthly temperatures is 7–25 °C [14].

Both areas contain several land-cover types representative for the Mediterranean area: natural oak woods with evergreen (*Quercus ilex* and *Quercus suber*) and deciduous (*Quercus cerris* and *Quercus frainetto*) species, broad-leaved mixed oaks forest, pasture, Mediterranean maquis, pseudo steppe, and mixed or pure forest of domestic pine (*Pinus pinea*) [15]. 

To cover different types of vegetation, five different sampling sites in CPE (site 1, 2, 3) and CNP (site 4, 5) have been chosen.

Site 1 (Casa del Pastore)—This site, located on the southwestern side of the Estate, is covered by a pine forest of *P. pinea* with trees up to 30 m high. The undergrowth is made up of sparse bushes of *Asparagus acutifolius*, *Laurus nobilis*, *Phillyrea latifolia* and *Rubus* spp. The herbaceous layer is very scarce and mainly formed by *C. distachya*, *Carex flacca* and *Poa trivialis*. A tree pasture with scattered specimens of *Q. suber* also characterises the site. The prevailing herbaceous species are annual-growing grasses, such as *Anthoxanthum odoratum*, *Briza maxima*, *Bromus mollis*, and *C. echinatus*. In addition, there are nitrophilous spiny species (*Cirsium strictum* and *Galactites tomentosa*) whose presence is due to the grazing of cattle [16]. A fallow area, characterised by annual growing grasses (in prevalence, *A. fatua*, *C. dactylon*, *Dasypyrum villosum*, *Lagurus ovatus*, and *P. trivialis*), completes the vegetation mosaic of the site [14].

Site 2 (Coltivi nord)—Situated in the North of the Estate, this site features a mosaic of vegetation characterised by low forest cover of *P. pinea*. Along the margins and clearings of this forest, in contact with pastures and crops, there are bushes of deciduous species (e.g., *Crataegus monogyna*, *Prunus spinosa*, *Cornus sanguinea*, *Clematis vitalba*, *Tamus communis*, and *Rubus ulmifolius*) mixed with evergreens, such as *P. latifolia*, *Rhamnus alaternus*, *Myrtus communis*, and *A. acutifolius*. 

Site 3 (Santola)—This wooded site, centrally located in the Estate, is mainly characterised by forest vegetation, with a prevalence of *Q. suber*, due to reforestation carried out after 1970 with native cork oak; lying on acidic sandy substrates, it is characterised by the presence in the underwood of evergreen shrubs (e.g., *P. latifolia*, *Ramnus alaternus*, *Cistus creticus*) and lianose shrubs, such as *Smilax aspera* and *Rubia peregrina*. 

Site 4 (Cerasella)—The site is characterised by the mesoigrophylus subcoastal oaks forest with *Q. frainetto* and *Q. cerris* referred to *Mespilo germanicae-Quercetum frainetto arbutetosum unedonis* phytocenosis. In the clearings caused by cutting and fire there are phytocoenosis with bushes of *Erica arborea*, *M. communis*, and *P. latifolia* [17,18].

Site 5 (Cocuzza)—This site is an internal gap of lowland oak forest with *Pruno-Rubion* mantle shrubs (*C. monogyna*, *Cistus creticus*, *Rubus* spp., *R. peregrina*, *A. acutifolius*, and *Hedera helix*). Herbaceous vegetation is dominated by annual herbs (e.g., *Cynosurus* spp., *Tuberaria guttata*, *B. maxima*, and *Coleostephus myconis*). It is included in the grassland of the *Helianthemion guttati* phytocenosis described for soils rich in siliceous sand of the subcoastal area of Latium [19].

### 2.2. Sampling and Analysis Procedures

To assess the relative frequencies of plant species, 25 permanent transects were utilised (five from each site). Sampling took place in the dry season (DS, May–August) and in the wet season (WS, November–February). Transects 50 m long were located to cover all the types of vegetation present in the study areas and were spaced by at least 100 m from each other. The quadrat method was used to assess plant frequency [20]: twenty-five samplings were carried out per transect, analysing 1 m^2^ of vegetation and skipping the following. Plant species were grouped into four vegetation forms: grasses (G), including in this form also graminoids; leguminous forbs (L); non-leguminous forbs (NLF); shrubs (S). The taxonomic nomenclature of the identified taxa followed Bartolucci et al. [21].

A plant from each observed species in the transepts was collected and processed according to the method described in Maia et al. [22]. In order to create a reference collection, histological fragments of each anatomical part were photographed by light microscopy and catalogued in a database using the image analyser Leica Q500IW (Leica Imaging System Ltd., Cambridge, UK). 

Faecal sampling took place monthly in the aforementioned periods along eight transepts (2 × 30 m) randomly distributed throughout each study site and distant at least 100 m from each other in order to reduce the probability to collect pellets from the same animal. All the collected pellets were fresh (bright brown faeces) and, for each collection, a minimum of six pellets, of various sizes and formats, were mixed to form a single composite sample. A total of 40 composites samples were analysed for *L. corsicanus* (8 months × 5 sites). Our consolidated experience in the microhistological technique made us prefer this method to others, perhaps faster (e.g., DNA metabarcoding) but also not without drawbacks [4,14]. 

Faecal pellets were processed according to the method described in Freschi et al. [11,14]. For each composite sample, 10 microscope slides were mounted. The slides were examined by light microscopy using the image analyser Leica Q500 IW, obtaining 200 readings for each sample, counting non-overlapping plant fragments in systematic transepts across a slide along alternate rows. Identification of plant species was performed by comparing the different characteristics of the epidermal cells and other structures (e.g., stomates and trichomes) with those of the plant reference collection built by collecting monthly the plants found in the study site. Microphotographs from all taxon/structures were made with the same magnification to facilitate a fast comparison between the reference collection and the faecal material.

This reference material is available at the Laboratory of Environmental and Applied Botany, University of Basilicata. Not identified fragments (6.7%) were classified as ‘unidentified’ and excluded from the analysis.

### 2.3. Statistical Analysis

Relative frequencies (*rf*) of plant species, families, and life forms were calculated by dividing the total number of fragments attributed to a given *taxon* by the total number of identified fragments. Data of the plant species identified in the study site were used to calculate the relative frequencies of each *taxon*, family, and vegetation form. Similarly, we calculated the relative frequencies of the plant species identified in the faeces by dividing the total number of fragments attributed to a given *taxon* by the total number of identified fragments [9,10,11,23,24]. 

Data of identified plant species composing the diet were also used to compute the following alpha diversity indices:

- Shannon diversity index (*H*) [25], whose value usually ranges between 1.5 and 3.5 and often does not exceed 4 [26];
- Margalef index (*D*) for species richness (higher the value the greater is the richness) [27];
- Buzas and Gibson evenness index (*E*) [28].

For each of the above indices, differences, between DS and WS were tested by Student’s *t*-test (*p* < 0.05).

To compare dietary similarity between DS and WS the Sørensen similarity index (*C_S_*) [29] was computed. *C_S_* index varies between 0 (no similarity) and 1 (complete similarity). 

Diet composition was analysed by multivariate analysis. Similarity matrices were constructed by using averages of the Bray-Curtis similarity coefficient [30]. Analysis of similarities (ANOSIM) was performed to test diet differences among sites using 999 permutations [31].

Diet selection was estimated for life forms and for shared plant families in vegetation and diet by Resource selection ratio (*w_i_*) [32]:

(1) $$w_{i}=\frac{oi}{pi}$$

where *o_i_* is the proportion of the botanical family (or life form) in the diet and *p_i_* is its available proportion (*w_i_* > 1, preference; *w_i_* = 1, indifference; *w_i_* < 1, avoidance). Differences were tested by *χ*^2^ test [33]. 

Data were analysed by R software (R Core Team, Wien, Austria) [34].

## 3. Results

### 3.1. Botanical Composition of the Sites in the Dry Season 

**Figure 2:**
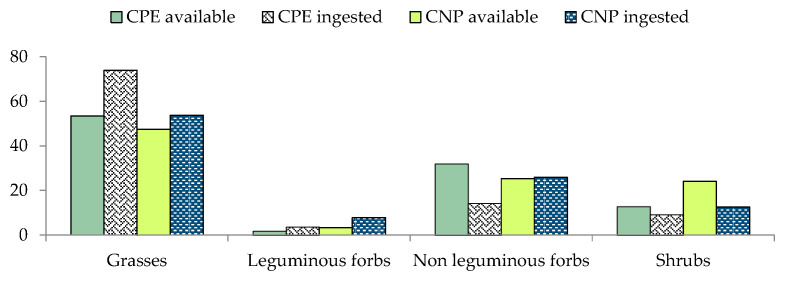
Percentage contribution of plant life forms in the vegetation (available) and in the diet (ingested), in dry season, in Castelporziano Presidential Estate (CPE) and Circeo National Park (CNP).

The most abundant life forms in CPE vegetation were grasses (53.37%) followed by non-leguminous forbs (32%), shrubs (12.81%), and leguminous forbs (1.82%) (Figure 2). In this site, 112 plant species belonging to 29 families were identified (Table A1 and Table A2). Regarding families, the most abundant were Poaceae (47.58%), Asteraceae (19.73%), and Rosaceae (5.11%). Among inventoried species the most representative were *Centaurea solstitialis* (5.13%), *D. villosum* (4.79%), *Lolium perenne* (4.23%), and *B. maxima* (4.20%) In CNP, as in in CPE, the most representative life form were grasses (47.48%), followed by non-leguminous forbs (25.35%), shrubs (24.04%), and leguminous forbs (3.36%) (Figure 2). In CNP, 95 species attributed to 33 families were identified. Poaceae was the most available family (40.54%), followed by Rosaceae (11.63%), Asteraceae (7.02%), and Lamiaceae (5.06%). The most abundant species were *C. distachya* (5.49%), *C. dactylon* (5.29%), and *B. sylvaticum* (4.79%) (Table A1 and Table A2). 

### 3.2. Botanical Composition of the Sites in the Wet Season 

**Figure 3:**
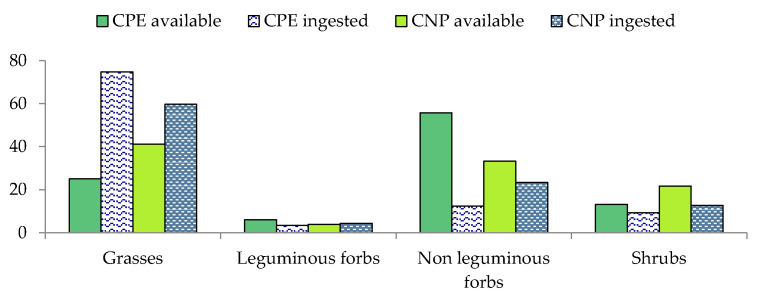
Percentage contribution of plant life forms in the vegetation (available) and in the diet (ingested), in wet season (DS), in Castelporziano Presidential Estate (CPE) and Circeo National Park (CNP).

In CPE the most abundant life form were non-leguminous forbs (55.8%), followed by grasses (25.05%), shrubs (13.17%), and leguminous forbs (6.07%) (Figure 3). On this site, 150 plant species, belonging to 43 families, were observed (Table A2 and Table A3). Over 57% of the observed species only belonged to four families: Poaceae (22.61%), Asteraceae (22.23%), Fabaceae (6.38%), and Geraniaceae (5.97%). The most representative species were *Cichorium intybus* (3.41%), *C. myconis* and *Picris hieracioides* (2.83% in both species), and *Hypochaeris radicata* (2.44%). In CNP, the most abundant life form resulted in grasses (41.11%), followed by non-leguminous forbs (33.25%), shrubs (21.73%), and leguminous forbs (3.85%) (Figure 3). The identified plant families and species were 33 and 108, respectively. The most representative families were Poaceae (28.15%), Asteracee (11.29%), Rosaceae (8.64%), and Cyperaceae (8.12%). The most frequent species were *C. distachya* (6.22%), *P. trivialis* (5.43%), *Clinopodium nepeta* (4.8%), and *Lolium arundinaceum* (4.55%) (Table A2 and Table A3).

### 3.3. Diet Composition in the Dry Season

In both sites, grasses were the most utilised life form in CPE (73.95%) and CNP (53.71%), followed by non-leguminous forbs (14.23% in CPE and 25.83% in CNP), shrubs (9.23% in CPE and 12.62 % in CNP), and leguminous forbs (3.71% in CPE and 7.74% in CNP) (Figure 2). In the diet of *L. corsicanus* from the Latium coast, 133 *taxa* belonging to 36 families were found (Table A1 and Table A2). Poaceae was the most representative family in the diet (63.7 % in CPE and 43.68% in CNP), followed by Asteraceae (7.13%), and Cyperaceae (5.61%) in CPE, Fabaceae (8.83%) and Asteraceae (6.82%) in CNP (Figure 2). The number of determined species was higher in CPE (103) than in CNP (96). In both sites, most of the *taxa* (71 in CPE and 68 in CNP) were ingested in low percentages (≤1%). Conversely, *B. sylvaticum*, *C. dactylon*, and *A. fatua* were among the most consumed species, together representing 17.01% and 16.26% of the diet in CPE and CNP, respectively (Table A1 and Table A2). 

### 3.4. Diet Composition in the Wet Season

Figure 3 shows, similarly to the dry period, that grasses was the most representative life form in the diet (74.77% in CPE and 59.69% in CNP), followed by non-leguminous forbs (12.47% in CPE and 23.32% in CNP), shrubs (9.36% in CPE and 12.65% in CNP), and leguminous forbs (3.4% and 4.37% in CPE and in CNP, respectively) (Figure 3). A total of 132 species belonging to 48 families were found in the wet season (Table A2 and Table A3). The number of species/families was 108/30 in CPE and 85/26 in CNP. The diet was composed mainly of Poaceae (60.84%), Amaryllidaceae (6.70%), and Cyperaceae (6.47%) in CPE and of Poaceae (48.76%), Fabaceae (11.6%), and Asteraceae (10.06%) in CNP. Among the inventoried species the most utilised in diet were *B. sylvaticum* (10.3%), *P. trivialis* (8.71%), and *C. distachya* (6.39%) in CPE, and *D. glomerata* (9.3%), *C. echinatus* (7.41%), and *S. junceum* (6.3%) in CNP (Table A2 and Table A3). 

### 3.5. Dietary Diversity and Similarity

Differences in DS vs. WS diet richness were observed only in CNP (*D*, 7.624 vs. 5.570, *p* = 0.029; *E*, 0.598 vs. 0.674, *p* = 0.021) (Table 1). In both sites, *Cs* similarity index showed a medium overlap among seasonal diets (0.677 in CPE and 0.569 in CNP).

**Figure A1:**
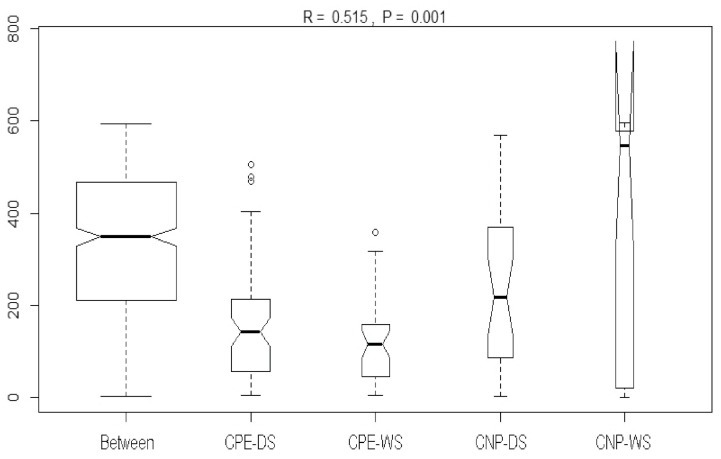
Analysis of similarities of diet in Castelporziano Presidential Estate, in dry (CPE-DS) and wet season (CPE-WS), and in Circeo National Park, in dry (CPE-DS) and wet season (CPE-WS).

ANOSIM analysis revealed that there were significant differences between sites in both DS and WS diets. Moreover, seasonal diets were significantly different in CNP (R = 0.515; *p* ≤ 0.001) (Figure A1).

### 3.6. Dietary Selectivity 

**Figure 4:**
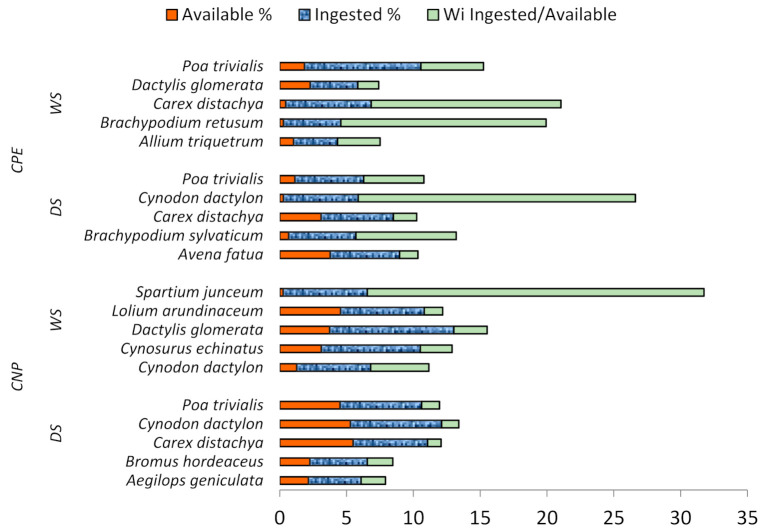
Incidence (%) in vegetation (Available), in diet (Ingested), and Selectivity (*W_i_*) of the most selected plant species in Castelporziano Presidential Estate and in Circeo National Park (CPE) in Dry (DS) and Wet season (WS).

Among the most abundant species in diets, those characterised by particularly high selectivity indices (*W_i_* > 2) are highlighted: *P. trivialis*, *C. distachya*, *Brachypodium retusum*, and *Allium triquetrum* (WS) and *P. trivialis*, *C. dactylon*, and *B. sylvaticum* (WS) in CPE; *Spartium junceum*, *D. glomerata*, *C. echinatus*, and *C. dactylon* (WS) in CNP (Figure 4).

During DS, in CPE, only the Poaceae family has been used more than expected according to its availability (Table 2). Conversely, Apiaceae, Asparagaceae, Asteraceae, Fagaceae, Geraniaceae, Malvaceae, Rhamnaceae, Rosaceae, and Rubiaceae were negatively selected. Instead, Amaryllidaceae, Asteraceae, Cyperaceae, and Poaceae, were positively selected in WS, and Apiaceae, Asteraceae, Brassicaceae, Caryophyllaeae, Fagaceae, Geraniaceae, Oleaceae, Rhamnaceae, Rosaceae, and Rubiaceae were avoided.

In CNP a positive selection was observed only in the wet period in Fabaceae and Poaceae (Table 3). Avoided families in both periods were Apiaceae, Asteraceae, Lamiaceae, and Oleaceae, while Cistaceae, Cyperaceae, and Rubiaceae were avoided only in WS.

**Figure A2:**
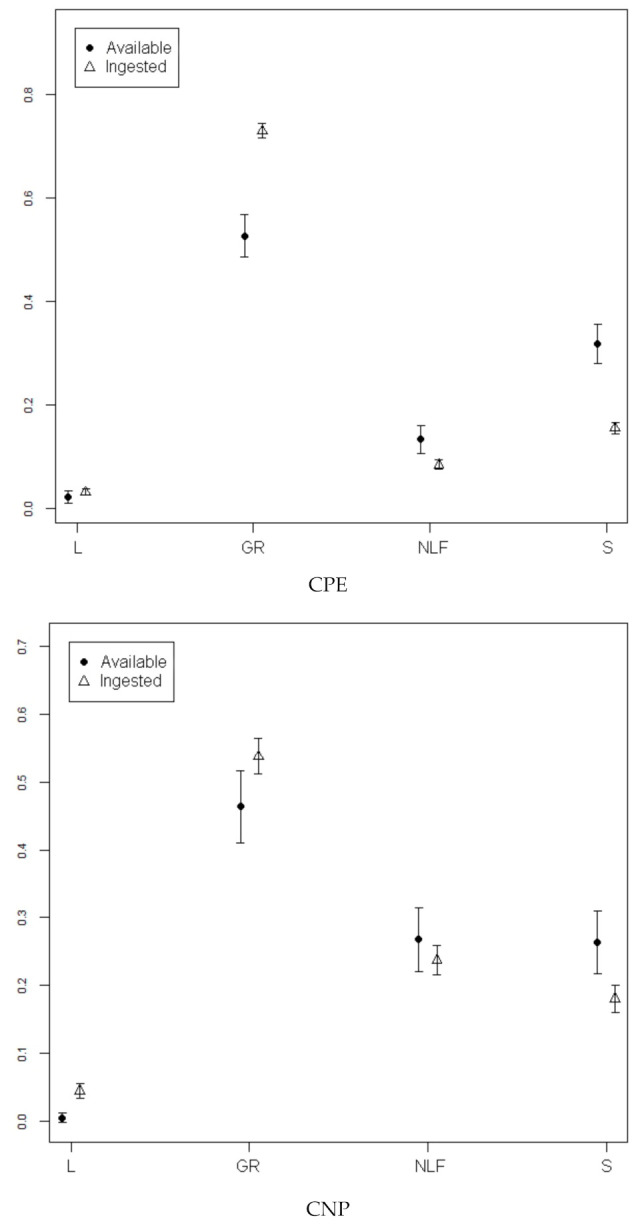
Annual selection (mean ± SD) on life forms in Castelporziano Presidential Estate (CPE) and in Circeo National Park (CNP). Grasses (GR), leguminous forbs (L), non-leguminous forbs (NLF), and shrubs.

Considering the annual selection of life forms, in both sites (Figure A2) grasses and leguminous forbs were preferred; conversely, non-leguminous forbs and shrubs were avoided.

## 4. Discussion

The main results emerging from our study were: (a) the wide feeding spectrum of the species, since it fed annually on 185 of the 229 plant species identified in vegetation; (b) the prevalence of grasses in CPE and in CNP, in DS and in WS diets, with the predominance of Poaceae, followed Cyperaceae, Amaryllidaceae, Asparagaceae, and Juncaeae, as other families of this life form; (c) the low number of plant species ingested at relatively high rates; (d) the plastic feeding behaviour of the Italian hare, as diet selectivity changed with the variety and abundance of food species. In the study sites, the most observed *taxa* were *C. dactylon*, *A. fatua* and *B. sylvaticum*. In particular, this last species is confirmed as an important constituent of the diet. High incidence in the diet of *Brachypodium* spp. was observed in studies conducted in the Basilicata region and in Corsica [4,9,10,11]. 

The preference for *Brachypodium* spp., also observed in ruminants [35], is probably linked to its wide distribution in various vegetation covers all year round. Considering Poaceae as a whole, their high contribution to the Italian hare’s diet could be motivated by their good palatability and high cellulose content, which can provide a useful reserve of energy [36]. 

Poaceae, Asteraceae and Fabaceae families constituted the bulk of the diet throughout the dry season. Similar preferences in diet were observed in the Italian hare in south Italy [12] and in Haute-Corse [4]. Castellaro et al. [36] underline the great importance of this group of plant species in the nutrition of herbivores with cecal fermentation, given the characteristics of their digestive system and the way in which nutrients are used. The increased palatability of forbs in the dry period could be attributed to their higher water and lower fibre contents in tissues in comparison with grasses [37,38]. Palatability was defined by Greenhalg and Reid [39] as the dietary characteristics that stimulate a selective response by the animal. Vallentine [40] cites, among the morphological and chemical factors that positively influence the palatability of a plant: the presence of succulent leaves, the absence of thorns, poor flowering, the accessibility to edible parts, the presence of young vegetative parts, the high content of protein and sugars, the low content of tannic substances that confer bitter taste, and the absence of alkaloids and glucosides with toxic action. On the other hand, the species which were normally avoided could be grazed on under compulsion due to the scarcity of food in the area. Concerning this observation, Asparagaceae, Amarillidaceae, and Cyperaceae which were avoided in CPE during DS were instead positively selected during WS. Moreover, CPE hares excluded *H. helix* and *Smilax aspera* from their diet, conversely CNP hares fed on these species even showing selectivity for *S. aspera* in WS. The lower availability of food herbaceous species determined by the dense canopy of CNP sampling sites could explain this feeding behaviour. Rubiaceae was used in small quantities and not selected. Conversely, in Corsica, this family was used more than expected according to its availability [4]. Overall, with the exception of Poaceae which was always preferred, we observe that feeding preferences of the Italian hare vary across different niches. Plants from this family represent the bulk of the diet also in *L. europaeus* [41,42,43,44,45,46,47,48], *Lepus timidus hibernicus* Bell, 1837 [49,50,51,52], *Lepus arcticus* Ross, 1819 [53], *Lepus californicus* Gray, 1837 [54,55,56,57], *Lepus flavigularis* Wagner, 1844 [58], *L. granatensis* [37], *Lepus starcki* Petter, 1963 [59]. In the present study grasses and non-leguminous forbs constituted a large portion of the diet of the Italian hare, while shrubs and leguminous forbs appeared to not be consumed in large quantities. Nevertheless, an underestimation of the incidence of these life forms in the diet could be related to their high digestibility. In Mediterranean environments, this underestimation could be lower in DS, when herbivores show a reduced digestibility of the dry matter of the selected plants [24]. 

Feeding preferences are very difficult to interpret and to understand as the factors involved vary spatially and in time, as well as to the availability and to relative abundance to associated species. 

In herbivores, several food strategies influence the rank-order selection of plants and their ingestion level at any given site in order to maximise energy intake, reduce energy expenditure or predation risks, or attenuate the toxic effects of plant secondary metabolites [60]. According to Shipley et al. [61], mammalian herbivores are considered generalists or specialists if the incidence of a family plant on diet is over or under 60%, respectively. These authors consider as facultative generalists to be the species in which the broad fundamental niche allows them to consume a wide variety of foods and that, occasionally, demonstrate a narrow realised niche, focused on less difficult plants than is the case with specialists. According to this definition, we can consider the Italian hare as a facultative generalist in its feeding strategy. Studies on feeding preferences of Brown hare [45,48] and Snowshoe hare [62] classified these species as predominantly generalist. Nevertheless, in these species, as in *L. corsicanus*, grasses are the main diet item even if with a declined importance in the dry season, when fibre content increases from early to late summer. In this period, in particular, the species could select some plant species that even if ingested in low quantities, would fulfil a nutritional role of production and a functional role as diet improvers [36].

## 5. Conclusions

Our study demonstrated that the diet of the Italian hare was characterised by a wide diversity of plant species in the dry and wet seasons. Nonetheless, the bulk of the diet consisted of a few species, among which the most abundant were *C. distachya*, *B*. *Sylvaticum*, and *C. dactylon.* Probably, the high selectivity toward these plants was also favoured by their high availability throughout the year. The significant differences in the composition of the diet–highlighted in the diversity indices–confirmed the great adaptability of the Italian hare to different niches and the influence of the vegetation on the feeding habits of the species. On the other hand, the wide spectrum of diet, besides reflecting the adaptation of the species to its habitat may be more beneficial to maintain the richness of species more so in environments characterised by high plant richness, such as our study sites. The Italian hare revealed its ecological plasticity highlighted by its capability for exploiting food resources, exhibiting an opportunistic behaviour in response to changes in their spatial and temporal availability.

## Figures and Tables

**Table 1:** Diet biodiversity indices (Mean±SE) in Castelporziano Presidential Estate (CPE) and Circeo National Park (CNP), and comparisons between dry season (DS) and wet season (WS).

Index	CPE			CNP		
	DS	WS	*p*	DS	WS	*p*
	Richness					
Shannon, *H*	3.252 ± 0.072	3.264 ± 0.084	0.917	3.176 ± 0.088	3.046 ± 0.102	0.340
Margalef, *D*	8.586 ± 0.481	8.640 ± 0.556	0.942	7.624 ± 0.589	5.570 ± 0.680	0.029
Buzas & Gibson, *E*	0.534 ± 0.017	0.538 ± 0.019	0.861	0.598 ± 0.020	0.674 ± 0.023	0.021
	Similarity					
Sorensen, *Cs*	0.677			0.569		

**Table 2:** Selection ratio (*w*_i_) on botanical families in Castelporziano Presidential Estate (CPE).

Family	CPE					
	DS			WS		
	*W_i_*	Feeding Behaviour	*p*-Value	*W_i_*	Feeding Behaviour	*p*-Value
Amaryllidaceae	4.386	I	0.064	3.115	P	0.002
Apiaceae	0.244	A	0.000	0.107	A	0.000
Asparagaceae	2.142	A	0.025	5.088	P	0.040
Asteraceae	0.341	A	0.000	0.205	A	0.000
Brassicaceae	8.183	I	0.386	0.297	A	0.000
Caryophyllaceae	0.442	I	0.074	0.015	A	0.000
Cistaceae	4.091	I	0.297	6.522	I	0.100
Cyperaceae	1.237	I	0.307	9.880	P	0.019
Fabaceae	1.894	I	0.071	0.485	A	0.000
Fagaceae	0.194	A	0.000	0.362	A	0.000
Geraniaceae	0.040	A	0.000	0.011	A	0.000
Malvaceae	0.398	A	0.007	0.104	A	0.000
Oleaceae	0.851	I	0.569	0.266	A	0.000
Plantaginaceae	0.752	I	0.515	1.380	I	0.675
Poaceae	1.316	P	0.000	2.259	P	0.000
Rhamnaceae	0.258	A	0.000	0.352	A	0.001
Rosaceae	0.314	A	0.000	0.605	A	0.000
Rubiaceae	0.663	A	0.066	0.440	A	0.000

Feeding behaviour: (P) preference, (I) indifference, (A) avoidance.

**Table 3:** Selection ratio (*w*_i_) on botanical families in Circeo National Park (CNP).

Family	CNP					
	DS			WS		
	*W_i_*	Feeding Behaviour	*p*	*W_i_*	Feeding Behaviour	*p*
Apiaceae	0.266	A	0.000	0.355	A	0.000
Asparagaceae	1.792	I	0.213	0.781	I	0.155
Asteraceae	0.654	A	0.003	0.741	A	0.011
Cistaceae	2.788	I	0.289	0.206	A	0.000
Cyperaceae	1.245	I	0.370	0.534	A	0.000
Fabaceae	15.087	I	0.189	3.157	P	0.011
Fagaceae	0.498	I	0.166	0.763	I	0.463
Juncaceae	4.929	I	0.272	0.548	I	0.083
Lamiaceae	0.525	A	0.000	0.034	A	0.000
Oleaceae	0.163	A	0.000	0.137	A	0.000
Poaceae	1.085	P	0.018	1.895	P	0.000
Rosaceae	0.289	A	0.000	0.304	A	0.000
Rubiaceae	1.195	I	0.742	0.123	A	0.000

Feeding behaviour: (P) preference, (I) indifference, (A) avoidance.

## Data Availability

Plant reference collection is available at the Laboratory of Environmental and Applied Botany, University of Basilicata, Potenza, Italy.

## Appendix A

**Table A1:** Dry season: frequencies (%) of Plant species, Families, and Life form (^1^) for CPE and CNP, in vegetation (available) and in diet (ingested).

Life Form	Family	Plant Species	CPE		CNP	
			Available	Ingested	Available	Ingested
Grasses	Amaryllidaceae	*Allium triquetrum*	0.81	3.62	0.24	1.74
	Asparagaceae	*Bellevalia romana*	0.06	0.23	0	0
		*Leopoldia comosa*	0.53	0.78	0	0
	Cyperaceae	*Carex distachya*	3.09	5.41	5.49	5.57
		*Carex echinata*	0.27	0.03	0	0
		*Carex flacca*	0.67	0.14	0.75	0.05
		*Carex hallerana*	0.27	0.03	0	0
	Juncaceae	*Juncus acutus*	0	0	0.3	2.62
		*Luzula forsteri*	0	0	0.15	0.05
	Poaceae	*Achnatherum bromoides*	2.91	2.94	1.03	1.25
		*Aegilops geniculata*	0.17	1.22	2.15	3.93
		*Alopercurus rendiei*	0.07	3.83	2	3.44
		*Anthoxanthum odoratum*	0.13	0.22	0.45	0.11
		*Arrhenatherum elatius*	3.49	0.75	0	0
		*Avena fatua*	3.78	5.19	0.5	1.91
		*Brachypodium pinnatum*	1.19	0.19	0	0
		*Brachypodium sylvaticum*	0.67	5.03	4.79	1.96
		*Briza maxima*	4.2	2.01	0	0
		*Briza media*	1.3	0.29	0	0
		*Briza minor*	0.01	0.03	0	0
		*Bromus hordeaceus*	1.81	3.74	2.25	4.3
		*Bromus racemosus*	1.13	2.21	0.31	0.49
		*Bromus sterilis*	2.01	2.46	0.23	0.87
		*Cynodon dactylon*	0.27	5.6	5.29	6.82
		*Cynosurus cristatus*	1.61	2.23	0.12	0.76
		*Cynosurus echinatus*	2.54	2.05	0	0
		*Dactylis glomerata*	0.13	2.41	3.44	2.4
		*Dactylis hispanica*	0.15	0.19	0.68	0.16
		*Dasypyrum villosum*	4.79	1.27	0.13	0.16
		*Elymus repens*	0.94	1.15	0	0
		*Lolium arundinaceum*	1.35	2.08	0.02	0.05
		*Festuca heterophylla*	0.14	0.19	0	0
		*Gastridium ventricosum*	1.13	1.28	3.12	3.71
		*Holcus lanatus*	0.05	0.6	2.5	0.71
		*Lagurus ovatus*	0.27	0.17	0.31	0.48
		*Lolium perenne*	4.23	2.42	0	0
		*Melica ciliata*	1.01	1.02	0.05	0.11
		*Oloptum miliaceum*	2.41	2.63	3.73	3.27
		*Phalaris minor*	1.12	1.52	0	0
		*Poa trivialis*	1.14	5.14	4.52	6.09
		*Sesleria autumnalis*	0	0	0.23	0.38
		*Setaria italica*	0	0	0.9	0.05
		*Vulpia myuros*	1.52	1.65	1.8	0.27
			53.37	73.95	47.48	53.71
Leguminous Forbs	Fabaceae	*Coronilla scorpioides*	0.14	0.63	0	0
		*Trifolium angustifolium*	0.13	1.48	0	0
		*Trifolium pratense*	1.01	0.93	1.53	2.23
		*Trifolium stellatum*	0.27	0.19	1.15	2.18
		*Vicia cracca*	0.27	0.48	0.68	3.33
			1.82	3.71	3.36	7.74
Non Leguminous Forbs	Apiaceae	*Daucus carota*	2.09	0.34	0	0
		*Foeniculum vulgare*	0.21	0.23	1.52	1.53
		*Smyrnium olusatrum*	0.13	0.01	0	0
	Asteraceae	*Bellis perennis*	0.07	0.16	0	0.05
		*Carthamus lanatus*	0.2	0	0	0
		*Centaurea solstitialis*	5.13	2.3	0	0.76
		*Chondrilla juncea*	0.12	0.49	0	0.11
		*Cichorium intybus*	2.96	0.31	0.7	1.64
		*Cirsium arvense*	2.15	0.29	2	0.16
		*Coleostephus myconis*	2.57	0.77	0	0
		*Crepis bursifolia*	0.08	0.12	0	0
		*Crepis leontodontoides*	0.1	0.2	2.33	0.33
		*Crepis neglecta*	0.03	0.14	0	0
		*Erigeron bonariensis*	0.13	0.7	1.22	1.25
		*Galactites tomentosa*	2.02	0	0	0
		*Helminthotheca echioides*	2.56	0	0	0
		*Hypochaeris achyrophorus*	0.13	0.12	0.36	0
		*Lactuca viminea*	0.01	0.03	0.1	0.34
		*Onopordon illyricum*	0.53	0	0	0
		*Picris echioides*	0.02	0.03	0	0
		*Picris hieracioides*	0.79	0.24	0	0
		*Reichardia picroides*	0.01	0.29	0.2	0.6
		*Rhagadiolus stellatus*	0.05	0.02	0	0
		*Senecio vulgaris*	0.02	0.65	0.08	0.11
		*Tanacetum* spp.	0.02	0.12	0.02	0.38
		*Urospermum picroides*	0.02	0.12	0.01	0.05
	Boraginaceae	*Buglossoides purpurocaerulea*	0.03	0.12	0.08	0.33
		*Cynoglossum* spp.	0.1	0	0	0
		*Echium vulgare*	0.13	0	0	0
		*Myosotis* spp.	0.12	0	0.6	0.16
		*Symphytum tuberosus*	0.02	0.02	0	0
		*Bunias erucago*	0.02	0.29	0	0
		*Capsella bursa pastoris*	0	0	0.4	0.05
		*Cardamine graeca*	0.02	0.63	0.05	0.22
		*Raphanus raphanistrum*	0.09	0.02	0	0
	Caryophyllaceae	*Cerastium arvense*	0	0	1.4	0
		*Lychnis flos-cuculi*	0	0	0.1	0.33
		*Silene alba*	0.13	0.07	0	0
		*Silene colorata*	0.24	0.05	0	0
		*Spergula pentrada*	0.02	0.07	0	0
		*Stellaria media*	0.01	0.02	0.05	0.16
		*Stellaria* spp.	0	0	0.05	0.06
	Chenopodiaceae	*Chenopodium album*	0.4	0.02	0	0
	Convolvulaceae	*Convolvulus arvensis*	0.02	0.03	0.24	0.44
	Dioscoreaceae	*Tamus communis*	0	0	0.51	0.87
	Dipsacaceae	*Smilax aspera*	1.61	0	0.55	0.65
	Celastraceae	*Euomymus latifolius*	0.27	0.92	0.25	0.27
	Euphorbiaceae	*Euphorbia amygdaloides*	0	0	0.95	1.31
		*Euphorbia helioscopia*	0	0	1.18	2.89
	Geraniaceae	*Geranium dissectum*	2.6	0.97	0.45	0.55
	Lamiaceae	*Mentha rotundifolia*	0.15	0.29	0.23	0.49
		*Phlomis herba venti*	0	0	1.11	3.22
		*Prunella vulgaris*	0.5	0.11	0.02	0.11
	Liliaceae	*Ornithogalum umbellatum*	0.27	1.19	0	0
	Malvaceae	*Malva sylvestris*	0.67	0.22	1.51	0.49
	Orobanchaceae	*Linaria vulgaris*	0	0	0.9	0.11
	Orobanchaceae	*Verbascum sinuatum*	0	0	0.45	0.71
	Plantaginaceae	*Plantago lanceolata*	0.4	0.65	0.96	0.05
		*Plantago media*	0.27	0.28	0	0
	Polygonaceae	*Rumex sanguineus*	0.41	0.02	0	0
	Primulaceae	*Lysimachia arvensis*	0.14	0.03	0	0
	Ranunculaceae	*Ranunculus repens*	1.07	0.15	1.58	2.02
	Rosaceae	*Sanguisorba minor*	0	0	1.81	0.38
	Rubiaceae	*Cruciata laevipes*	0	0	0.23	0.05
		*Galium palustre*	0	0	0.25	0.49
		*Rubia peregrina*	0.14	0.38	0.2	0.93
		*Sherardia arvensis*	0	0	0.45	1.07
	Zygophyllaceae	*Tribulus terrestris*	0	0	0.25	0.11
			32	14.23	25.35	25.83
Shrubs	Aceraceae	*Acer campestre*	0	0	0.39	0.49
	Araliaceae	*Hedera helix*	0.94	0	2.25	0.55
	Asparagaceae	*Asparagus acutifolius*	1.75	2.69	0.45	3.16
		*Ruscus aculeatus*	0	0	1.58	0.82
	Cistaceae	*Cistus creticus*	0.25	2.11	0.44	0.69
	Fabaceae	*Cytisus hirsutus*	0.33	0.15	0.09	1.09
	Fagaceae	*Quercus cerris*	0	0	0.45	0.05
		*Quercus ilex*	0	0	0.08	0.05
		*Quercus suber*	1.52	0.52	0.15	0.23
		*Quercus virgiliana*	0.13	0.11	0	0
	Hypolepidaceae	*Pteridium aquilinum*	0	0	0.23	0.05
	Lamiaceae	*Calamintha nepeta*	0.21	0.26	3.02	0.05
		*Teucrium chamaedrys*	0	0	0.68	0.55
	Myrtaceae	*Myrtus communis*	0	0	0.9	0.11
	Oleaceae	*Fraxinus ornus*	0	0	1.4	0.16
		*Olea europaea*	0	0	0.57	0.22
		*Phyllirea latifolia*	1.75	1.04	0.51	0.38
	Rhamnaceae	*Rhamnus alaternus*	0.81	0.27	1.03	0.65
	Rosaceae	*Crataegus monogyna*	0.81	0.28	1.5	0.76
		*Prunus spinosa*	0.4	0.26	2.03	0.27
		*Pyrus amygdaliformis*	0.01	0.26	0.51	0.38
		*Rosa canina*	1.2	0.23	0.23	0.33
		*Rubus ulmifolius*	2.69	1.02	4.52	0.54
		*Sorbus torminalis*	0.01	0.03	1.03	1.04
			12.81	9.23	24.04	12.62

**Table A2:** Frequencies (%) of Families in Castelporziano Presidential Estate (CPE) and in Circeo National Park (CNP), in vegetation (available) and in diet (ingested).

Family	Dry Season				Wet Season			
	CPE		CNP		CPE		CNP	
	Available	Ingested	Available	Ingested	Available	Ingested	Available	Ingested
Aceraceae	0	0	0.39	0.49	0	0	0.03	0.14
Amaryllidaceae	0.81	3.62	0.24	1.74	1.81	6.7	3.58	3.9
Apiaceae	2.43	0.58	1.52	1.53	4.24	0.54	2.34	0.91
Araceae	0	0	0	0	4.25	0	0	0
Araliaceae	0.94	0	2.25	0.55	0.06	0	0.42	0
Asparagaceae	2.34	3.69	2.03	3.98	0.55	3.33	5.87	5.49
Asphodelaceae	0	0	0	0	0.08	0.31	1.7	0
Asteraceae	19.73	7.13	7.02	6.82	22.23	5.42	11.29	10.06
Boraginaceae	0.4	0.14	0.68	0.49	0.63	0	0.02	0.38
Brassicaceae	0.13	0.94	0.45	0.27	2.96	1.08	0.85	0,00
Caprifoliaceae	0	0	0	0	0	0	0.42	0
Caryophyllaceae	0.4	0.2	1.6	0.55	0.47	0.04	0.01	0.53
Celastraceae	0.27	0.92	0.25	0.27	0	0	0	0
Chenopodiaceae	0.4	0.02	0	0	0.16	0	0	0.05
Cistaceae	0.25	2.11	0.44	0.69	0.31	2.44	0.42	0,00
Convolvulaceae	0.02	0.03	0.24	0.44	0.1	0.12	0	0.1
Cyperaceae	4.3	5.61	6.24	5.62	0.55	6.47	8.12	0
Dioscoreaceae	0	0	0.51	0.87	0	0	0	6.26
Dipsacaceae	1.61	0	0.55	0.65	0	0	0	0
Ericaceae	0	0	0	0	0	0	0.85	0
Euphorbiaceae	0	0	2.13	4.2	4.25	0.35	0.42	0.67
Fabaceae	2.15	3.86	3.45	8.83	6.38	3.45	4.6	11.6
Fagaceae	1.65	0.63	0.68	0.33	2.46	1.08	1.27	1.88
Gentianaceae	0	0	0	0	0.05	0.77	0	0
Geraniaceae	2.6	0.97	0.45	0.55	5.97	0.08	1.27	1.88
Hypericaceae	0	0	0	0	0.08	0	0	0
Hypolepidaceae	0	0	0.15	0.05	0	0	0	0
Iridaceae	0	0	0	0	0.08	0.23	1.27	0,00
Juncaceae	0	0	0.45	2.67	0.08	0.77	1.27	0.77
Lamiaceae	0.86	0.66	5.06	4.42	1.41	0.31	6.29	0.29
Lauraceae	0	0	0	0	0.08	0	0	0
Liliaceae	0.27	1.19	0	0	0	0	0.21	0
Malvaceae	0.67	0.22	1.51	0.49	0.63	0.08	0.01	0.05
Myrtaceae	0	0	0.9	0.11	0	0	1.49	0
Oleaceae	1.75	1.04	2.48	0.76	2.2	0.7	2.54	0.38
Orobanchaceae	0	0	1.35	0.82	1.02	0	0	0
Papaveraceae	0	0	0	0	0.24	0	0	0
Pinaceae	0	0	0	0	0.24	0	0	0
Plantaginaceae	0.67	0.93	0.96	0.05	0.24	0.39	0.42	3.22
Poaceae	47.58	63.7	40.54	43.68	22.61	60.84	28.15	48.76
Polygonaceae	0.41	0.02	0	0	3.61	0.08	0	0
Portulacaceae	0	0	0	0	0.02	0.08	0	0
Primulaceae	0.14	0.03	0	0	0.08	0	0.42	0.24
Ranunculaceae	1.07	0.15	1.58	2.02	1.18	0.15	2.12	0.14
Rhamnaceae	0.81	0.27	1.03	0.65	0.63	0.27	0	0.14
Rosaceae	5.11	2.05	11.63	2.66	4.09	2.94	8.64	1.64
Rubiaceae	0.14	0.38	1.13	2.54	1.25	0.66	3.19	0.43
Scrophulariaceae	0	0	0	0	0.08	0.31	0.42	0
Smilacaceae	0	0	0	0	1.18	0	0.06	0.14
Solanaceae	0	0	0	0	1.18	0.01	0	0
Urticaceae	0	0	0	0	0.16	0	0	0
Zygophyllaceae	0	0	0.25	0.11	0	0	0	0

**Table A3:** Wet season: frequencies (%) of Plant species, Families, and Life form (^1^) in Castelporziano Presidential Estate (CPE) and in Circeo National Park (CNP), in vegetation (available) and in diet (ingested).

Life Form	Family	Plant Species	CPE		CNP	
			Available	Ingested	Available	Ingested
Grasses	Amaryllidaceae	*Allium polyanthum*	0.53	2.09	0.6	0.19
		*Allium subhirsutum*	0.25	1.32	2.42	3.22
		*Allium triquetrum*	1.03	3.29	0.56	0.48
	Cyperaceae	*Carex distachya*	0.45	6.39	6.22	3.85
		*Carex flacca*	0.08	0.04	1.55	2.17
		*Carex remota*	0.02	0.04	0.35	0.24
	Juncaceae	*Juncus acutus*	0.08	0	0.42	0.77
		*Luzula forsteri*	0	0.77	0.85	0
	Poaceae	*Alopecurus rendlei*	0.11	1.67	0.01	0.58
		*Anisantha sterilis*	0.21	0.27	0	0
		*Aristella bromoides*	0.52	1.43	0.02	0.1
		*Arrhenatherum elatius*	0.36	1.16	0.04	0
		*Avena barbata*	1.31	3.1	0.3	0.43
		*Brachypodium phoenicoides*	0	0	0.94	1.83
		*Brachypodium pinnatum*	0.02	0.04	0	0
		*Brachypodium retusum*	0.28	4.3	0	0
		*Brachypodium sylvaticum*	1.96	10.3	1.91	4.57
		*Briza maxima*	1.49	2.67	0.29	0.58
		*Briza media*	0.15	0.12	0	0
		*Briza minor*	0.62	0.85	0	0
		*Bromus hordeaceus*	0.25	0.77	0.21	0.34
		*Cynodon dactylon*	1.02	3.02	1.27	5.53
		*Cynosurus cristatus*	0.96	1.55	0	0
		*Cynosurus echinatus*	0.79	0.89	3.11	7.41
		*Dactylis glomerata*	2.28	3.56	3.73	9.3
		*Dactylis hispanica*	0.75	1.36	0	0
		*Dasypyrum villosum*	1.69	2.2	0.63	0.87
		*Festuca heterophylla*	0.2	0.15	0.42	0.96
		*Festuca myuros*	0.69	1.43	1.7	2.07
		*Holcus lanatus*	0.2	0.93	0.23	1.15
		*Lagurus ovatus*	0.07	0.04	0.88	0.76
		*Lolium arundinaceum*	1.12	1.05	4.55	6.27
		*Lolium perenne*	0.4	0.54	1.68	0.38
		*Lolium rigidum*	0.95	1.32	0	0
		*Melica ciliata*	0.28	2.36	0	0
		*Oloptum miliaceum*	0.02	0.04	0	0
		*Phleum nodosum*	0	0	0.54	0.29
		*Poa pratensis*	1	1.9	0	0
		*Poa trivialis*	1.86	8.71	5.43	1.93
		*Sesleria autumnalis*	0.23	0.66	0.25	0.1
		*Setaria italica*	0.3	0.04	0	0
		*Triticum vagans*	0.52	2.4	0	3.32
			25.05	74.77	41.11	59.69
Leguminous forbs	Fabaceae	*Coronilla scorpioides*	0	0	0.2	0.29
		*Hippocrepis biflora*	0.55	0	0.99	0
		*Lathyrus aphaca*	0.08	0.35	0.21	0
		*Medicago arabica*	1.1	0.15	0.15	0.19
		*Medicago orbicularis*	1.64	0.23	0.32	0.77
		*Trifolium angustifolium*	0.4	0.35	1.06	1.54
		*Trifolium campestre*	0	0	0.35	0.14
		*Trifolium pratense*	0.16	1.08	0	0
		*Trifolium repens*	0.71	0.5	0	0
		*Trifolium resupinatum*	0.03	0.04	0	0
		*Trifolium stellatum*	0.36	0.35	0.12	0.19
		*Trifolium vesiculosum*	0	0	0.45	1.25
		*Vicia cracca*	0.96	0.08	0	0
		*Vicia* spp.	0.08	0.27	0	0
			6.07	3.4	3.85	4.37
Non Leguminous Forbs	Apiaceae	*Chaerophyllum* spp.	1.81	0	0	0
		*Daucus carota*	1.41	0.54	1.36	0.87
		*Foeniculum vulgare*	0.63	0	0.98	0.05
		*Oenanthe pimpinelloides*	0.24	0	0	0
		*Tordylium apulum*	0.16	0	0	0
	Araceae	*Arum italicum*	2.36	0	0	0
		*Biarum tenuifolium*	1.89	0	0	0
	Asparagaceae	*Bellevalia romana*	0.02	0.66	1.54	1.4
		*Muscari comosum*	0.08	0.08	2.06	3.18
	Asphodelaceae	*Asphodelus ramosus*	0.08	0.31	1.7	0
	Asteraceae	*Anthemis arvensis*	2.09	0	1.27	0.29
		*Bellis perennis*	1.02	0.43	0	0
		*Carlina vulgaris*	0	0	0.12	0.14
		*Centaurea solstitialis*	0.96	0.62	0	0
		*Chondrilla juncea*	0.48	0.23	0	0
		*Cichorium intybus*	3.41	0.39	0.23	0.48
		*Cirsium arvense*	1.18	0.04	0	0
		*Coleostephus myconis*	2.83	0.15	0	0
		*Crepis bursifolia*	0.24	0.08	0	0
		*Crepis leontodontoides*	0.52	0.15	1.51	0.19
		*Crepis neglecta*	0.08	0.58	1.7	0.1
		*Dittrichia viscosa*	0.24	0.39	0	0
		*Erigeron bonariensis*	0.24	1.51	0	0
		*Helminthotheca echioides*	1.1	0	0.65	0.82
		*Hyoseris radiata*	0	0	0.31	0.67
		*Hypochaeris radicata*	2.44	0.19	0.64	1.78
		*Lactuca viminea*	0.08	0.04	0.54	0.1
		*Picris hieracioides*	2.83	0.04	3.12	5.49
		*Ptilostemon strictus*	1.73	0	0	0
		*Reichardia picroides*	0.08	0.08	1.2	0
		*Senecio vulgaris*	0.08	0.43	0	0
		*Sonchus oleraceus*	0.63	0.08	0	0
	Boraginaceae	*Borago officinalis*	0.63	0	0	0
		*Buglossoides purpurocaerulea*	0	0	0.02	0.38
	Brassicaceae	*Bunias erucago*	0.08	0.04	0	0
		*Cardamine graeca*	0.47	1.05	0	0
		*Diplotaxis tenuifolia*	1.31	0	0.85	0
		*Raphanus raphanistrum*	0.86	0	0	0
		*Sinapis arvensis*	0.24	0	0	0
	Caprifoliaceae	*Sixalix atropurpurea*	0	0	0.21	0.14
	Caryophyllaceae	*Cerastium arvense*	0.08	0	0	0
		*Silene alba*	0.39	0.04	0	0
		*Stellaria media*	0	0	0.01	0.05
	Chenopodiaceae	*Beta vulgaris*	0.16	0	0	0
	Convolvulaceae	*Convolvulus arvensis*	0.1	0.12	0	0
	Euphorbiaceae	*Euphorbia amygdaloides*	1.52	0.35	0.21	0.24
		*Euphorbia helioscopia*	1.89	0	0.16	0
		*Euphorbia peplis*	0.84	0	0.05	0.43
	Gentianaceae	*Centaurium erythrarea*	0.05	0.77	0	0
	Geraniaceae	*Erodium cicutarium*	1.96	0	0	0
		*Geranium dissectum*	0.71	0	0	1.68
		*Geranium robertianum*	2.36	0	0	0
		*Geranium rotundifolium*	0.94	0.08	1.27	0.19
	Hypericaceae	*Hypericum perforatum*	0.08	0	0	0
	Iridaceae	*Hermodactylus tuberosus*	0.03	0.08	0	0
		*Romulea bulbocodium*	0.05	0.15	1.27	0
	Lamiaceae	*Ajuga reptans*	0	0	0.21	0
		*Lamium album*	0.31	0	0.64	0
		*Mentha suaveolens*	0	0.23	0	0.19
		*Stachys romana*	0	0.08	0	0.1
		*Salvia verbenaca*	0.31	0	0.21	0
		*Stachys sylvatica*	0	0	0.42	0
	Linaceae	*Linum* spp.	0	0	0.21	0
	Malvaceae	*Malva sylvestris*	0.63	0.08	0.01	0.05
	Oxalidaceae	*Oxalis corniculata*	1.02	0	0	0
	Papaveraceae	*Papaver rhoeas*	0.24	0	0	0
	Plantaginaceae	*Linaria vulgaris*	0.03	0.19	0	0
		*Plantago argentea*	0	0	0.21	0
		*Plantago lanceolata*	0.02	0.19	0	3.22
		*Plantago media*	0.03	0	0	0
		*Veronica serpyllifolia*	0.16	0	0.21	0
	Polygonaceae	*Polygonum aviculare*	1.57	0	0	0
		*Rumex bucephalophorus*	0.16	0	0	0
		*Rumex conglomeratus*	0.39	0	0	0
		*Rumex obtusifolius*	0.08	0.04	0	0
		*Rumex sanguineus*	1.41	0.04	0	0
	Portulacaceae	*Portulaca trituberculata*	0.02	0.08	0	0
	Primulaceae	*Cyclamen repandum*	0.05	0	0.21	0
		*Lysimachia arvensis*	0.03	0	0.21	0.24
	Ranunculaceae	*Ficaria verna*	0	0	0.85	0
		*Ranunculus repens*	0.66	0.12	0	0
		*Ranunculus monspeliacus*	0	0	0.57	0
	Rosaceae	*Agrimonia eupatoria*	0.32	0.74	0.21	0.43
		*Poterium sanguisorba*	0	0	2.49	0
	Rubiaceae	*Cruciata laevipes*	0.39	0	0.64	0.14
		*Galium aparine*	0.5	0	0.56	0.14
		*Rubia peregrina*	0.36	0.66	0.98	0.14
		*Sherardia arvensis*	0	0	1.01	0
	Scrophulariaceae	*Verbascum blattaria*	0.06	0	0	0
		*Verbascum sinuatum*	0.02	0.31	0.42	0
	Solanaceae	*Solanum nigrum*	1.18	0.01	0	0
	Urticaceae	*Urtica dioica*	0.16	0	0	0
			55.8	12.47	33.25	23.32
Shrubs	Aceraceae	*Acer campestre*	0	0	0.03	0.14
	Araliaceae	*Hedera helix*	0.06	0	0.42	0
	Asparagaceae	*Asparagus acutifolius*	0.37	2.13	0.64	0.87
		*Ruscus aculeatus*	0.08	0.46	1.63	0.05
	Caprifoliaceae	*Lonicera etrusca*	0	0	0.21	0.38
	Cistaceae	*Cistus creticus*	0.31	2.44	0.42	0.1
	Ericaceae	*Arbutus unedo*	0	0	0.42	0
	Ericaceae	*Erica arborea*	0	0	0.42	0
	Fabaceae	*Cytisus hirsutus*	0	0	0.14	0.29
		*Cytisus scoparius*	0.16	0	0.36	0.63
		*Spartium junceum*	0.16	0.04	0.25	6.3
	Fagaceae	*Quercus ilex*	0.21	0.15	0.54	1.68
		*Quercus pubescens*	0	0	0.57	0
		*Quercus suber*	2.25	0.93	0.16	0.19
	Lamiaceae	*Clinopodium nepeta*	0.79	0	4.8	0
	Lauraceae	*Laurus nobilis*	0.08	0	0	0
	Myrtaceae	*Myrtus communis*	0	0	1.49	0
	Oleaceae	*Fraxinus ornus*	0	0	2.33	0
		*Olea europaea*	0.93	0.43	0	0.05
		*Phyllirea latifolia*	1.27	0.27	0.21	0.34
	Pinaceae	*Pinus pinea*	0.24	0	0	0
	Ranunculaceae	*Clemantis flammula*	0.52	0.04	0.7	0.14
	Rhamnaceae	*Rhamnus alaternus*	0.63	0.27	0	0.14
	Rosaceae	*Crataegus monogyna*	0.47	0.5	0.21	0.24
		*Prunus spinosa*	0.16	0.89	1.06	0.05
		*Pyrus communis*	0.08	0	0.94	0.19
		*Rosa canina*	0.52	0.5	0.42	0.1
		*Rubus ulmifolius*	1.58	0.04	1.45	0
		*Sorbus torminalis*	0.96	0.27	1.85	0.63
	Smilacaceae	*Smilax aspera*	1.18	0	0.06	0.14
	Viburnaceae	*Viburnum tinus*	0.16	0	0	0
			13.17	9.36	21.73	12.65

## References

[1] Angelici F.M., Galli A., Petrozzi F. (2011). The Apennine hare *Lepus corsicanus* in Latium, Central Italy: A habitat suitability model and comparison with its current range. Hystrix.

[2] Riga F., Trocchi V. IUCN Red List of Threatened Species. http://www.iucn.it/scheda.php?id=-1247663548.

[3] Pietri C., Alves P.C., Melo-Ferreira J. (2011). Hares in Corsica: High prevalence of *Lepus corsicanus* and hybridation with introduced *L. europaeus* and *L. granatensis*. Eur. J. Wildl. Res..

[4] Rizzardini G., Fascetti S., Pietri C., Riga F., Cosentino C., Freschi P. (2019). Feeding preferences in dry season of the Italian hare (*Lepus corsicanus*) in two sites of Corsica. Eur. J. Wildl. Res..

[5] Trocchi V., Riga F. (2001). Piano D’azione Nazionale per la Lepre Italica (Lepus Corsicanus). Quaderni di Conservazione Della Natura.

[6] Freschi P., Fascetti S., Riga F., Cosentino C., Rizzardini G., Musto M. (2017). Diet composition of the Italian roe deer (Capreolus capreolus italicus) (Mammalia: Cervidae) from two protected areas. Eur. Zool. J..

[7] Duffy J.E., Cardinale B.J., France K.E., McIntyre P.B., Thébault E., Loreau M. (2007). The functional role of biodiversity in ecosys-tems: Incorporating trophic complexity. Ecol. Lett..

[8] De Battisti R., Migliore S., Masutti L., Trocchi V. The diet of the Italian hare *Lepus corsicanus* on Etna Mountain, Sicily. Proceedings of the Abstract Book of the 2nd World Lagomorph Conference.

[9] Freschi P., Fascetti S., Musto M., Mallia E., Blasi A.C., Cosentino C., Paolino R. (2014). Diet of the Apennine hare in a southern Italy Regional Park. Eur. J. Wildl. Res..

[10] Freschi P., Fascetti S., Musto M., Mallia E., Cosentino C., Paolino R. (2014). Diet of the Italian hare (*Lepus corsicanus*) in a semi-natural landscape of southern Italy. Mammalia.

[11] Freschi P., Fascetti S., Musto M., Cosentino C., Paolino R., Valentini V. (2016). Seasonal variation in food habits of the Italian hare in a south Apennine semi-natural landscape. Ethol. Ecol. Evol..

[12] Buglione M., Maselli V., Rippa D., De Filippo G., Trapanese M., Fulgione D. (2018). A pilot study on the application of DNA metabarcoding for non-invasive diet analysis in the Italian hare. Mamm. Biol..

[13] Buglione M., Petrelli S., De Filippo G., Troiano C., Rivieccio E., Notomista T., Maselli V., Di Martino L., Carafa M., Gregorio R. (2020). Contribution to the ecology of the Italian hare (*Lepus corsicanus*). Sci. Rep..

[14] Freschi P., Fascetti S., Riga F., Rizzardini G., Musto M., Cosentino C. (2021). Feeding Preferences of the Italian Roe Deer (*Capreolus capreolus italicus* Festa, 1925) in a Coastal Mediterranean Environment. Animals.

[15] Della Rocca B., Pignatti S., Mugnoli S., Bianco P.M. (2001). La Carta della Vegetazione della Tenuta di Castelporziano. Il Sistema ambientale Della Tenuta Presidenziale di Castelporziano.

[16] Freschi P., Musto M., Paolino R., Cosentino C., Vastola A. (2015). Grazing and biodiversity conservation: Highlights on a natura 2000 network site. The Sustainability of Agro-Food and Natural Resource Systems in the Mediterranean Basin.

[17] Anzalone B., Lattanzi E., Lucchese F., Padula M. (1997). The vascular flora of the Circeo National Park (Parco Nazionale del Circeo-Lazio). Webbia.

[18] Blasi C., Stanisci A., Filesi L., Milanese A., Perinelli E., Riggio L. (2002). Syndinamics of lowland Quercus frainetto and Quercus cer-ris forests in Lazio. Fitosociologia.

[19] Lucchese F., Pignatti S. (1990). Sguardo sulla vegetazione del Lazio marittimo. Quad. Accad. Naz. Dei Lincei.

[20] Bonham C.D. (1989). Measurements for Terrestrial Vegetation.

[21] Bartolucci F., Peruzzi L., Galasso G., Albano A., Alessandrini A., Ardenghi N.M.G., Astuti G., Bacchetta G., Ballelli S., Banfi E. (2018). An updated checklist of the vascular flora native to Italy. Plant Biosyst. Int. J. Deal. All Asp. Plant Biol..

[22] Maia M., Rego F., Machado F.S. (2003). Determining optimal sampling schemes to study red deer diets by fecal analysis. Silva Lusit..

[23] Garnick S., Barboza P.S., Walker J.W. (2018). Assessment of Animal-Based Methods Used for Estimating and Monitoring Rangeland Herbivore Diet Composition. Rangel. Ecol. Manag..

[24] Orellana C., Parraguez V.H., Arana W., Escanilla J., Zavaleta C., Castellaro G. (2019). Use of Fecal Indices as a Non-Invasive Tool for Nutritional Evaluation in Extensive-Grazing Sheep. Animals.

[25] Shannon C.E., Weaver W. (1964). The Mathematical Theory of Communication.

[26] Margalef R. (1972). Interpretation not strictly statistical of representation of biological entities in multifactorial space. Investig. Pesq..

[27] Margalef R. (1958). Information theory in ecology. Gen. Syst..

[28] Buzas M.A., Gibson T.G. (1969). Species diversity: Benthonic foraminifera in western North Atlantic. Science.

[29] Sørensen T.A. (1948). A method of establishing groups of equal amplitude in plant sociology based on similarity of species content, and its application to analyses of the vegetation on Danish commons. Biol. Skr. Dan. Vid. Sel..

[30] Clarke K.R. (1993). Non-parametric multivariate analyses of changes in community structure. Aust. J. Ecol..

[31] Warton D.I., Wright S.T., Wang Y. (2012). Distance-based multivariate analyses confound location and dispersion effects. Methods Ecol. Evol..

[32] Manly B.F.L., McDonald L., Thomas D.L., McDonald T.L., Erickson W.P. (2002). Resource Selection by Animals: Statistical Design and Analysis for Field Studies.

[33] Zar J.H. (1999). Biostatistical Analysis.

[34] R Core Version 3.6.1. https://www.R-project.org/.

[35] Fedele V., Pizzillo M., Claps S., Morand-Fehr P., Rubino R. (1993). Grazing behaviour and diet selection of goats on native pasture in Southern Italy. Small Rumin. Res..

[36] Castellaro G., Orellana C., Escanilla J. (2021). Summer Diet of Horses (*Equus ferus caballus* Linn.), Guanacos (*Lama guanicoe* Müller), and European Brown Hares (*Lepus europaeus* Pallas) in the High Andean Range of the Coquimbo Region, Chile. Animals.

[37] Paupério J., Alves P.C. (2008). Diet of the Iberian hare (Lepus granatensis) in a mountain ecosystem. Eur. J. Wildl. Res..

[38] Kuijper D.P.J., van Wieren S.E., Bakker J.P. (2004). Digestive strategies in two sympatrically occurring lagomorphs. J. Zool..

[39] Greenhalgh J.F.D., Reid G.W. (1971). Relative palatability to sheep of straw, hay and dried grass. Br. J. Nutr..

[40] Vallentine J.F. (2012). Grazing Management.

[41] Frylestam B. (1986). Agricultural land use effects on the winter diet of Brown Hares (*Lepus europaeus* Pallas) in southern Sweden. Mammal Rev..

[42] Chapuis J.L. (1990). Comparison of the diets of two sympatric lagomorphs, *Lepus europaeus* (Pallas) and *Oryctolagus cuniculus* (L.) in an agroecosystem of the Ile-de-France. Z. Säugertierkd.

[43] Wray S. (1992). The Ecology and Management of European Hares (*Lepus europaeus*) in Commercial Coniferous Forestry. Ph.D. Thesis.

[44] Reichlin T.S., Klansek E., Hacklaender K. (2006). Diet selection by hares (*Lepus europaeus*) in arable land and its implications for habitat management. Eur. J. Wildl. Res..

[45] Puig S., Videla F., Cona M.I., Monge S.A. (2007). Diet of the brown hare (*Lepus europaeus*) and food availability in northern Patagonia (Mendoza, Argentina). Mamm. Biol..

[46] Kontsiotis V., Tsiompanoudis A.C., Bakaloudis D.E. (2011). The influence of habitat structure on the European brown hare *Lepus europaeus* food habits in mountainous areas of northern Greece. Mammalia.

[47] Green K., Davis N.E., Robinson W., McAuliffe J., Good R.B. (2013). Diet selection by European hares (*Lepus europaeus*) in the alpine zone of the Snowy Mountains, Australia. Eur. J. Wildl. Res..

[48] Sokos C., Andreadis K., Papageorgiou N. (2015). Diet adaptability by a generalist herbivore: The case of brown hare in a Mediterranean agroecosystem. Zool. Stud..

[49] Hewson R., Hinge M.D.C. (1990). Characteristics of the Home Range of Mountain Hares Lepus timidus. J. Appl. Ecol..

[50] Tangney D., Fairley J., O’Donnell G. (1995). Food of Irish hares *Lepus timidus hibernicus* in western Connemara, Ireland. Acta Thériol..

[51] Wolfe A., Whelan J., Hayden T.J. (1996). The diet of the mountain hare (*Lepus timidus hibernicus*) on coastal grassland. J. Zool..

[52] Dingerkus S.K., Montgomery W.I. (2001). The diet and landclass affinities of the Irish hare *Lepus timidus hibernicus*. J. Zool..

[53] Klein D.R., Bay C. (1994). Resource partitioning by mammalian herbivores in the high Arctic. Oecologia.

[54] Uresk D.W. (1978). Diets of the Black-Tailed Hare in Steppe Vegetation. Rangel. Ecol. Manag..

[55] Johnson R.D., Anderson J.E. (1984). Diets of Black-Tailed Jack Rabbits in Relation to Population Density and Vegetation. Rangel. Ecol. Manag..

[56] Hoagland D.B. (1992). Feeding ecology of an insular population of the black-tailed jackrabbit (Lepus californicus) in the Gulf of Cali-fornia. Southwest Nat..

[57] Wansi T., Pieper R.D., Beck R.F., Murray L.W. (1992). Botanical content of black-tailed jackrabbit diets on semidesert rangeland. Great Basin Nat..

[58] Lorenzo C., Carrillo-Reyes A., Gómez-Sánchez M., Velázquez A., Espinoza E. (2011). Diet of the endangered Tehuantepec jackrabbit, Lepus flavigularis. Therya.

[59] Mekonnen T., Yaba M., Bekele A., Malcolm J. (2011). Food Selection and Habitat Association of Starck’s Hare (Lepus starcki Petter, 1963) in the Bale Mountains National Park, Ethiopia. Asian J. Appl. Sci..

[60] Hartmann T., Rosenthal G.A., Berenbaum M.R. (1991). Alkaloids. Herbivores: Their Interactions with Secondary Plant Metabolites.

[61] Shipley L.A., Forbey J.S., Moore B.D. (2009). Revisiting the dietary niche: When is a mammalian herbivore a specialist?. Integr. Comp. Biol..

[62] Seccombe-Hett P., Turkington R. (2008). Summer diet selection of snowshoe hares: A test of nutritional hypotheses. Oikos.

